# Supplementary figures and images for: Hybrid whale algorithm with evolutionary strategies and filtering for high-dimensional optimization: Application to microarray cancer data
Source: PLoS One. 2024 Mar 11;19(3):e0295643. doi: 10.1371/journal.pone.0295643 (PMC10927076; doi:10.1371/journal.pone.0295643)

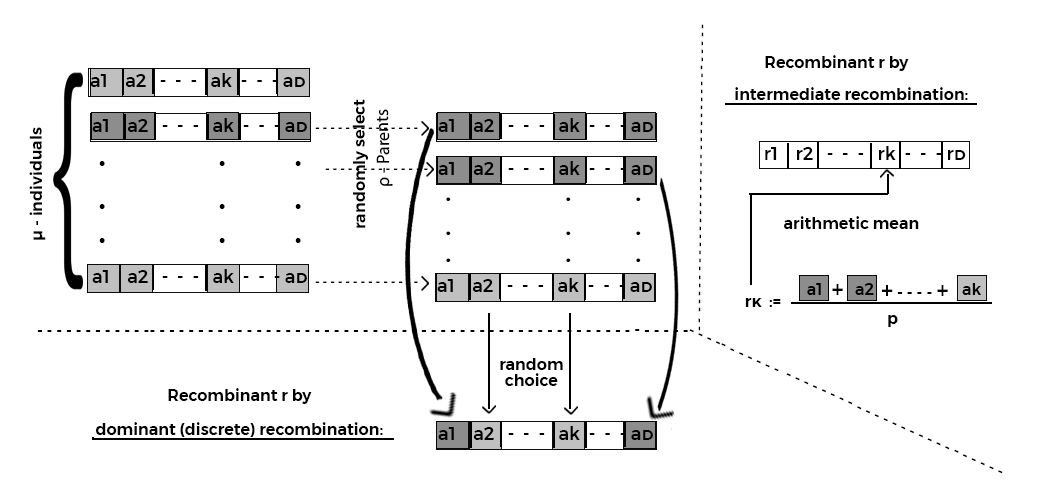

Supplement: S1 Fig — (TIF) [file pone.0295643.s001.tif]
