# Supplementary material for: Hybrid whale algorithm with evolutionary strategies and filtering for high-dimensional optimization: Application to microarray cancer data
Source: PLoS One. 2024 Mar 11;19(3):e0295643. doi: 10.1371/journal.pone.0295643 (PMC10927076; doi:10.1371/journal.pone.0295643)
Supplement: Attachment — Submitted filename: Response to reviewers.docx [file pone.0295643.s002.docx]

**Manuscript**

**Response to reviewers**

Dear Dr. Omar,

Thank you for allowing us to submit a revised draft of the manuscript "Hybrid Whale Algorithm with Evolutionary Strategies and Filtering for High-Dimensional Optimization: Application to Microarray Cancer Data" for publication in the *Plos One.* We appreciate the time and effort you and the reviewers dedicated to providing feedback on our manuscript and are grateful for the insightful comments and valuable improvements to our paper. We have incorporated most of the suggestions made by the reviewers. Please see below, in blue, for a point-by-point response to the reviewers' comments and concerns. All page numbers refer to the revised manuscript file with tracked changes.

**Reviewers' Comments to the Authors:**

**Response to Reviewer #1**

i. Large number of English language issues, including in abstract. Needs extensive review by technical editor proficient in English. for example, the very first sentence is missing an 'IS' 'The standard whale algorithm IS easily trapped in suboptimal and high-dimensional regions.

It is corrected by using Grammarly English correction software.

ii. What is meant by trapped in subotimal regions? do you mean suboptimal solutions?

Trapped in suboptimal regions" refers to a phenomenon in optimization where an algorithm or search process becomes stuck in a local minimum or maximum of a function rather than finding the global minimum or maximum.

Suboptimal solutions refer to any solutions that are not the best for a given optimization problem. When an algorithm becomes trapped in a suboptimal region, it can only find suboptimal solutions and cannot explore the entire search space to find the optimal solution. Yes, that's correct. Suboptimal regions mean suboptimal solutions.

iii. How is the algorithm trapped in high-dimensional regions? do you mean high dimensionality problems create problems finding optimal solutions?

In high-dimensional optimization problems, the number of possible combinations of parameters increases exponentially with the number of variables, making it challenging to find the global optimum. As a result, many optimization algorithms can become trapped in suboptimal regions when working in high-dimensional spaces.

One reason algorithms can become trapped in high-dimensional regions is due to the curse of dimensionality. In high-dimensional spaces, the volume of the space increases exponentially with the number of dimensions, making it difficult to explore the search space efficiently. For example, the distance between two randomly chosen points becomes larger, and the number of points required to get good coverage of the space increases exponentially with the dimensionality.

Another reason algorithms can become trapped in high-dimensional regions is the sparsity of the search space. In high-dimensional spaces, the objective function is typically dominated by a few influential dimensions, and most other dimensions are either irrelevant or redundant. This makes it difficult for an algorithm to explore the relevant dimensions and identify the global optimum.

Yes, that's correct. High-dimensional optimization problems, where the number of variables or dimensions is large, can make it challenging to find the optimal solution. As the number of dimensions increases, the search space grows exponentially, and the algorithms have to explore many combinations of parameters, which becomes computationally expensive. Additionally, the curse of dimensionality can make it challenging to explore the search space efficiently, leading to the possibility of the algorithm becoming trapped in suboptimal regions.

As a result, finding the global optimum in high-dimensional optimization problems can be challenging, and the solution obtained may only be suboptimal.

Are there more references that could be used in section 2.2?

added

iv. The WOA is not well explained; more background is needed.

Some additional background information on the Whale Optimization Algorithm (WOA) on pages 158-180 has been included.

v. The recombinative strategy description around line 167 is not well described at all and appears to mix generalities with specific parameters (5 parents, 100 offsping) that are of unclear origin.

We have revised the manuscript per your recommendations and added more clarity on the recombinative strategy, and the relevant changes have been made on lines 182-205.

vi. An intuitive sense of the contrast between the collaborative nature of WOA and the recombination would be helpful. As I understand it, the WOA typically probabilistically updates each search agent; sometimes this uses the influence of a randomly selected search agent by using the influence of other search agents. The recombination is not clear as described in lines 154-174; the best I can understand is that the initial positions are found by recombinative methods rather than randomly, but it is not well described.

Yes, you are right. The initial positions are found by discrete recombinative methods rather than randomly. A detailed description regarding the initial population has been provided in the revised manuscript, lines 207-222 and 258-270. We have also provided the recombination strategy details on lines 78-99 and 182-205.

vii. The presentation order is very hard to follow. The WOA is finally described in more detail starting at line 197, along with the updated version around line 220, but there is still no explanation of the number of parents / offspring which appear to be hard coded to some arbitrary number "100".

We have rearranged the presentation order of the paper in the revised manuscript for a better understanding. Furthermore, Regarding the number of parents/offspring used in the recombination process, the authors chose a hyperparameter based on empirical studies. We choose

$\mu=100$ random population and $\lambda=100$ offspring

viii. The solution appears to simply be selecting the initial population with the recombinative method, but it is not clear why this is better than simply having a better 'initial guess'.
ix. The choice of the initial population can significantly impact the performance of a metaheuristic algorithm. In some cases, it may be beneficial to use a recombinative method to generate the initial population because it can help to explore the search space more thoroughly and generate diverse solutions. The recombinative process is perfect when the search space is large or complex, and it may not be easy to generate a diverse set of initial solutions manually. On the other hand, if the search space is relatively small or straightforward, it may be sufficient to use a few well-chosen initial solutions as the starting population. In such cases, a recombinative method may not provide any additional benefit and could waste computational resources. We have been provided with the literature review for the diversity impact of the initial population and why this is better than simply having a better 'initial guess on lines 207-222.
x. what is the origin of the test functions in section 2.4.1?

It is added on lines No. 279-285 in the revised manuscript.

xi. I do like the concept but are these functions that are used commonly in optimization problems particularly with WOA?

Yes, the functions commonly used in optimization problems can be used in the Whale Optimization Algorithm (WOA) as well. One of the advantages of using WOA is that it can be applied to a wide range of optimization problems that involve different types of objective functions.

xii. If they were cited earlier than line 244, it would be helpful to repeat this ag line 244 "13 international test functions" (and what, exactly, is 'international' about a 'test function'?)

We have corrected it on lines No. 28,281,324,480. To clarify, the phrase "13 international test functions" in line 244 refers to a set of benchmark functions that are commonly used to evaluate the performance of optimization algorithms. These functions are referred to as "international" because they have been widely recognized and adopted by the research community around the world.

xiii. The table 1 says 'mean' and the document (line 247) says "Avg".

Corrected

xiv. The benchmark column is 0 for everything except F8 (Schwefel's function)for dim=30 and dim=100 which is very confusing, because those are both 418.9829*5 (is that supposed to be an exponent, like 10e5?)

The benchmark column for Schwefel's function ($F_{8}$) is not 0 for all dimensions.

The correct information is that the global minimum value of Schwefel's function ($F_{8}$) is 418.9829 * n, where n is the dimension of the function. For example, if n = 30, the global minimum value is 12,569.487, and if n = 100, the global minimum value is 41,898.29.

xv. Section 3 appears to jump into the use of data sets with cancer sets; I assume "filtration" refers to dimensionality reduction but this is very confusiong.

We have rearranged the whole manuscript for clarity. Yes, filtration refers to dimensionality reduction, and we corrected it in the revised manuscript on pages 335-382.

xvi. Section 4 follows with cancer datasets, which are apparently generalized as 'high dimensional data'.

You are right, but we revised it as cancer data on line 335 for clarity.

xvii. In particular it is not clear to me why the recombination is helpful beyond providing a better initial answer for the WOA as opposed to a bounded random set of selections.

Recombination is used in many evolutionary algorithms, including the WOA, to generate new solutions by combining information from multiple parent solutions. In the case of the WOA, the recombination operation is used to generate a new candidate solution by combining the information from two randomly selected parent solutions. The main advantage of recombination is that it can exploit the information contained in the parent solutions to generate potentially better offspring than the parent solutions themselves. Offspring can inherit beneficial traits from both parent solutions and combine them more effectively than randomly selecting solutions from a bounded set. In the context of the WOA, the recombination operation can help to generate a more diverse set of candidate solutions that explore the search space more effectively. It can lead to a better chance of finding high-quality solutions to the optimization problem, especially if the initial set of solutions is limited or poorly suited to the problem.

xviii. If that is the main contribution, I would likely recommend rejection.

The paper's main contribution has been added on lines 121-141.

**Response to reviewer # 2**

i. There are numerous grammatical errors in the manuscript. I am not an editor so I did not comprehensively list every such error, but I strongly insist that the authors go through the manuscript and correct all such errors. (I took the time to point out these errors in Abstract and a bit in the Intro)

Thank you for your valuable feedback on our manuscript. We appreciate your concern regarding the grammatical errors in the manuscript. We want to inform you that we have used a grammar checker tool like Grammarly to help us identify and correct the errors. However, we understand that no automated tool is perfect and may not catch all the errors. We have reviewed the manuscript and made all the necessary corrections. We have also considered the assistance of a professional editor or proofreader to help us further improve the quality of the manuscript.

ii. The title is quite wordy and difficult to understand. Perhaps shorten it?

It has been corrected in the revised manuscript.

iii. The capitalization is also inconsistent (e.g. "state-of-the-art" and "high dimensionality" should be capitalized).

Corrected; see lines 82-481 and Table 8, column No. 1.

Abstract

iv. Line 24: grammar "The standard whale algorithm [is] easily trapped..."

corrected

v. Line 25-26: grammar "The computer-generated initial populations [are] generally unevenly distributed..."

corrected

vi. Line 27-28: grammar "A fusion of this algorithm based on ... [is] proposed."

corrected

vii. Line 30: assess the "complexity" of what?

Corrected

viii. Line 33: Sentence fragment?

corrected

Introduction

ix. Line 38: What does "these" refer to?

Corrected; See line 38 on the revised manuscript.

x. Line 39: "genetic engineering. (1)." -> "genetic engineering (1)."

Corrected; See line 39

xi. Line 41: "Started" -> "Start"

Corrected

xii. You are describing a generic algorithm, so use the present tense. The past tense implies that you are referring to a specific run of an algorithm in the past.

Corrected

xiii. Line 42: "optimal answers"? If they are already optimal, why continue the search?

Corrected

xiv. Line 44: "Genetic algorithm" -> "Genetic algorithms"

Corrected

xv. How are evolutionary strategies different from genetic algorithms? Also, you write as though there is a singular unique genetic algorithm. Isn't it more a family of algorithms? Same for evolutionary algorithms.

Evolutionary strategies and genetic algorithms are members of the broader family of evolutionary algorithms, which are a class of metaheuristic optimization algorithms inspired by biological evolution. Although they share some similarities, these two approaches have some key differences.

One key difference is how they handle the representation of the solution space. Genetic algorithms typically use a binary string or string of integers to represent the solutions, which can be manipulated through crossover and mutation operations to generate new candidate solutions. Evolutionary strategies, on the other hand, typically use a continuous vector representation of the solutions, which can be modified through mutation and recombination.

Another difference is in how they perform the selection. Genetic algorithms typically use selection methods such as roulette wheel selection or tournament selection, where individuals with higher fitness values are more likely to be selected for reproduction. In contrast, evolutionary strategies typically use a form of selection called (1+1)-ES, where a single individual is mutated to produce a new candidate solution. Only the better of the two solutions is retained.

Regarding your second question, you are correct that genetic algorithms and evolutionary algorithms are more accurately described as families of algorithms rather than singular, unique algorithms. Some many variations and modifications can be made to the basic algorithmic structure to improve performance or adapt to different problem domains. The same is true for evolutionary strategies and other types of evolutionary algorithms.

xvi. Line 53: What's "WOA"?

Corrected, Whale Optimization Algorithm (WOA)

xvii. Line 58-60: "Using gene expression profiles to identify and classify malignant and normal tissues is the most difficult application of machine learning" This statement is overly broad. Many would strongly disagree, such as those working with brain data.

Corrected; see lines 101-104

xviii. Line 63: sentence fragment "The Support Vector Machine (SVM), which is widely used in machine learning models (12)."
xix. Corrected; see lines 106-108
xx. How are SVMs relevant/related to your work? How is it related to DNA microarray classification? Otherwise this section seems a bit random/out-of-place.

We have used mean square error (MSE) as the objective function and SVM as the prediction modal. We minimized the MSE incorporating it into the Whale optimization algorithm and the proposed algorithm as the objective function. Additionally, we optimized the SVM parameter with minimum MSE. Regarding DNA microarray classification, SVMs are commonly used to analyze and classify gene expression data. DNA microarrays allow researchers to simultaneously measure the expression levels of thousands of genes, producing high-dimensional data that can be challenging to analyze and interpret using traditional statistical methods. SVMs can be applied to these data sets to identify patterns and classify samples from different disease states, such as cancerous or non-cancerous tissues.

In the paper context, SVMs have been used as a benchmark method for the proposed fusion algorithm. We have compared the performance of our fusion algorithm to that of an SVM or other commonly used machine learning algorithm to demonstrate its superiority.

xxi. Line 70: You already said this earlier.

Corrected

xxii. Line 77: "Tumour" -> "tumour" Also, you use both "tumor" and "tumour." Pick one and maintain consistency throughout the manuscript.

Corrected

xxiii. Line 90: Who/what is the "operator of the algorithm"?
xxiv. Corrected; typo mistake.
xxv. Line 91: I thought you were using WOA as the optimization algorithm. How are you simultaneously using SVM?
xxvi. Support Vector Machine (SVM) has been proven to perform much better when dealing with high-dimensional datasets and numerical features. Although SVM works well with the default value, the performance of SVM can be improved significantly using parameter optimization. We applied two methods which are the recombinative evolutionary strategy hybrid whale algorithm (RESHWOA)and Whale optimization algorithm (WOA), to optimize the SVM parameters. Our experiment showed that SVM parameter optimization using RESHWOA always finds near-optimal parameter combinations within the given ranges. However, WOA was not; therefore, it was reliable only in low-dimensional datasets with few parameters. SVM parameter optimization using RESHWOA can be used to solve the problem of high dimensional regions. RESHWOA has proven to be more stable than WOA. The average running time on 6 datasets shows that RESHWOA was almost better than WOA.
xxvii. Furthermore, the RESHWOA's results were slightly better than the grid search in 5 of 6 datasets. For more clarity, the whale optimization algorithm has been used to optimize the SVM's hyperparameter with minimum mean square error. WOA is the optimizer, SVM is the prediction modal, and MSE is the objective function.
xxviii. Line 95: What is an "operator"?

Corrected; Typo mistake. See lines 47-48 and 92-94 for more details.

xxix. Line 96: Font size changes?

Corrected

xxx. Line 99: Missing period.

corrected

xxxi. Line 104: "calculated" -> "organized"

Corrected

**Section 2**

xxxii. Line 112-113: Decapitalize "Hybrid algorithm techniques"

Corrected

xxxiii. Line 118: Decapitalize "Logistic chaotic mapping"

Corrected; see line 72

xxxiv. Is the algorithm called RESHWOA or RESWOA? What does it stand for?

The algorithm called Recombinative evolutionary strategy hybrid whale optimization algorithm (RESHWOA)

xxxv. Section 2.1 should go first in section 2, as it provides necessary background for the reader to understand your method.

Corrected; see lines 146,157,181,206.

xxxvi. Section 2.2 has different font.

corrected

xxxvii. Line 137-138: What is a decision variable? What are the parents? This section is missing significant exposition/background.

Decision variables are the offspring generated through a discrete recombination strategy. Parents are the individuals selected through reproduction. Background of the evolutionary strategy has been added; see lines 78-99,182-205.

xxxviii. Section 2.2: How does the actual recombination take place, operationally? What is the reprsenentation of the "DNA"? How does mutation occur, operationally?

We added a new S1 Fig. 18, demonstrating how the recombination occurs operationally. For the second part of the question, the representation of DNA in microarray cancer data by measuring gene expression levels using specific probes that target individual genes. In the discrete recombination strategy, offspring are selected randomly, so no mutation occurs operationally.

xxxix. Line 159: What is the "dominant ρ recombination"?

See S1 Fig. 18

xl. Equation 1.1: What is the "random" function? Is it sampling an element uniformly at random from a given set?

Yes, it is sampling an element uniformly randomly from a given set.

xli. Line 170: Different citation style? (square bracket vs parentheses)

Corrected

xlii. You have described how recombination occurs, but how does mutation occur

No mutation occurs operationally in the discrete recombination strategy.

xliii. Lines 177-179: This is the second equation but is labeled (1). What is the "rand" function? Why is there a subscript outside of "random(...)"? Is this position update not dependent on the previous position X_(i)?

We have formatted these equations and updated them in the new format. See line 201.

xliv. Lines 180-181: Add commas to separate the clauses.

Updated

xlv. Lines 182-192: What are these equations?

Updated see line 201

xlvi. Equation 2: What is C? What is X_(*)(i)? How does it differ from X(i)?

A and C are coefficient vectors, X* is the position vector of the best solution obtained so far, and X is the position vector. See lines 137-137

xlvii. Equation 3: Why is this update equation different from equation (1)?
xlviii. Equation 4: What is r_1?

$r_{1}$ randomly generated vector lies between 0 and 1.

xlix. Equation 6: This is the third distinct equation for X(i+1).

Yes, you are right; Actually, equations 3,6 and 9 show the three different behaviours of the whales, Encircling Prey Behavior if $p<0.5$, Attacking Prey Behavior if $p\geq0.5$ and Searching Prey Behavior if $\left| A \right|<1$ or $\left| A \right|>1$. According to these equations, whales update their behaviours depending on the values of p and A. For more details, check the reference given below.

Mirjalili S, Lewis A. The Whale Optimization Algorithm. Adv Eng Softw [Internet]. 2016;95:51–67. Available from: http://dx.doi.org/10.1016/j.advengsoft.2016.01.008

l. Equation 8: D' is never used anywhere else. What is X_(rand)(i)? How does it differ from X(i)?

$D^{'}$ Indicates the distance of the ith whale to the prey, and it is only part of equation 6. Where $X_{\left( rand \right)}(i)$ is a random whale position; X (i) is the position vector.
