# Supplementary material for: Hybrid whale algorithm with evolutionary strategies and filtering for high-dimensional optimization: Application to microarray cancer data
Source: PLoS One. 2024 Mar 11;19(3):e0295643. doi: 10.1371/journal.pone.0295643 (PMC10927076; doi:10.1371/journal.pone.0295643)
Supplement: Attachment — Submitted filename: Response to the reviewers.docx [file pone.0295643.s003.docx]

**Manuscript**

**Response to reviewers**

Dear Dr. Omar,

Thank you for allowing us to submit a second revised draft of the manuscript "Hybrid Whale Algorithm with Evolutionary Strategies and Filtering for High-Dimensional Optimization: Application to Microarray Cancer Data" for publication in the *Plos One.* We appreciate the time and effort you and the reviewers dedicated to providing feedback on our manuscript and are grateful for the insightful comments and valuable improvements to our paper. We have incorporated most of the suggestions made by the reviewers. Please see below, in blue, for a point-by-point response to the reviewers' comments and concerns. All page numbers refer to the revised manuscript file with tracked changes.

**Reviewers' Comments to the Authors:**

**Response to Reviewer #1:**

 Please use additional screening for grammar and punctuation, such as Microsoft Word (there are still many cases of missing spaces and other simple errors, but overall the paper is improved from before).

Thank you for your valuable feedback on our paper. We sincerely appreciate your efforts in reviewing our work and providing constructive suggestions. We are glad to know that the paper has shown improvement since the previous version.

Regarding your concern about grammar and punctuation, we acknowledge the importance of ensuring the highest quality of language in our paper. To address this, we have taken the following steps:

1. **Using Microsoft Word:** We have run the entire document through the Microsoft Word spelling and grammar check. This process has helped us identify and correct many instances of missing spaces and other simple errors.
2. **Professional Proofreading:** Additionally, we have enlisted the assistance of professional proofreaders to meticulously review the paper. Their expertise has been instrumental in catching and rectifying any remaining grammar and punctuation issues.
3. **Multiple Revisions:** Throughout the revision process, we have paid extra attention to refining the language, sentence structure, and punctuation. We have carefully combed through the text to ensure coherence and clarity.
4. **Using Grammarly:** In addition to carefully proofreading the content, we have also utilized Grammarly software to enhance the clarity and correctness of the text. This combination of human proofreading and Grammarly assistance has helped us address any spelling and grammar errors, ensuring a more polished and accurate presentation of our work.

While we have taken significant steps to improve the paper's language, we understand that achieving absolute perfection is crucial. Therefore, we will perform another thorough review to make certain that all grammar and punctuation errors are effectively addressed.

**Reviewer #3:**

The manuscript has been significantly improved and in this form is of considerable scientific interest. I believe that the authors of the article managed to prove a significant advantage of the combined RESHWOA method over the classical Whale Optimization Algorithm (WOA). I believe that the discrete recombination (DR) strategy can be used to improve a number of other algorithms

Thank you for your positive and encouraging feedback on our manuscript. We are delighted to learn that you find the improved version to be of considerable scientific interest. Your insightful comments and appreciation for our work are truly motivating.

Regarding your observation on the combined RESHWOA method, we are pleased that you recognize the significant advantage it offers over the classical Whale Optimization Algorithm (WOA). We put considerable effort into designing and implementing the RESHWOA approach, and we are thrilled that our efforts have yielded promising results.

Your suggestion of using the discrete recombination (DR) strategy to enhance other algorithms is indeed thought-provoking. We wholeheartedly agree with your assessment and believe that the DR strategy's versatility could be applicable to various optimization algorithms beyond our current research. We intend to explore further the potential of the DR strategy and its broader implications in the optimization domain. In our future work, we plan to investigate its effectiveness in combination with other metaheuristic algorithms, aiming to contribute to the advancement of optimization techniques.

We are pleased to inform you that we have taken your suggestion to heart and incorporated it into our paper. In the conclusion section, specifically on line 495.

**Additional Editor Comments:**

In its current state, the level of English throughout the manuscript needs improvement. You may wish to ask a native speaker to check your manuscript for grammar, style, and syntax.

All the corrections have been done.

**Journal Requirements:**

Please review your reference list to ensure that it is complete and correct. If you have cited papers that have been retracted, please include the rationale for doing so in the manuscript text, or remove these references and replace them with relevant current references. Any changes to the reference list should be mentioned in the rebuttal letter that accompanies your revised manuscript. If you need to cite a retracted article, indicate the article’s retracted status in the References list and also include a citation and full reference for the retraction notice.

All references in the manuscript have been reviewed and updated to comply with PLOS ONE referencing style. We have not cited any retracted papers in our work.
