# Supplementary material for: Hybrid whale algorithm with evolutionary strategies and filtering for high-dimensional optimization: Application to microarray cancer data
Source: PLoS One. 2024 Mar 11;19(3):e0295643. doi: 10.1371/journal.pone.0295643 (PMC10927076; doi:10.1371/journal.pone.0295643)
Supplement: Attachment — Submitted filename: Respose to the Reviewers.docx [file pone.0295643.s004.docx]

**Manuscript**

**Response to reviewers**

**Dear Dr. Omar,**

Thank you for allowing us to submit a 3rd revised draft of the manuscript "Hybrid Whale Algorithm with Evolutionary Strategies and Filtering for High-Dimensional Optimization: Application to Microarray Cancer Data" for publication in the *Plos One.* We appreciate the time and effort you and the reviewers dedicated to providing feedback on our manuscript and are grateful for the insightful comments and valuable improvements to our paper. We have incorporated most of the suggestions made by the reviewers. Please see below, in blue, for a point-by-point response to the reviewers' comments and concerns. All page numbers refer to the revised manuscript file with tracked changes.

**Reviewers' Comments to the Authors:**

**Response to Reviewer #4:**

All the concerns have been addressed well; I thus recommend this manuscript to be published in Plos one.

Thank you very much for your positive feedback and for recommending the publication of our manuscript in PLOS ONE. We appreciate your time and effort in reviewing our work, and we're delighted to hear that all your concerns have been addressed satisfactorily.

Your recommendation is highly valuable to us, and we look forward to sharing our research with the scientific community through PLOS ONE. Your feedback has contributed significantly to the improvement of our manuscript, and we are grateful for your constructive comments.

**Response to Reviewer #5:**

The paper, titled "Hybrid Whale Algorithm with Evolutionary Strategies and Filtering for High-Dimensional Optimization: Application to Microarray Cancer Data," submitted as PONE-D-23-01358R2, has shown significant improvement from the initial draft. However, several aspects still require further enhancement, with a notable need for an extended literature review.

The revised version of the paper demonstrates commendable progress in terms of content. The authors have refined their algorithm and provided a more comprehensive explanation of the proposed Hybrid Whale Algorithm with Evolutionary Strategies and Filtering. This has resulted in increased clarity regarding the methodology used for high-dimensional optimization in the context of microarray cancer data analysis.

The paper has made significant strides in explaining the Hybrid Whale Algorithm, making it more accessible to a wider readership. The authors have successfully addressed some of the ambiguities present in the previous draft, clarifying the key concepts and steps involved in the algorithm.

The presentation of empirical results has also been improved, with more detailed analysis and visualization of outcomes in the context of microarray cancer data. This contributes to a better understanding of the algorithm's performance and its potential applications.

We sincerely appreciate your thoughtful and comprehensive review of our manuscript. Your feedback has been immensely valuable in guiding us toward further improvements in our work. We are pleased to hear that you have observed significant progress in the revised version of our paper. Your observation regarding the need for an extended literature review is duly noted, and we will certainly work on enhancing this aspect of the manuscript. We understand the importance of providing a comprehensive background to contextualize our research better.

The fact that you found our refined algorithm explanation to be more accessible and the clarification of key concepts as commendable is encouraging. We are committed to ensuring that the methodology is explained in a clear and concise manner to benefit a broad readership.

Furthermore, your feedback on the presentation of empirical results is well received. We acknowledge the importance of providing a thorough analysis and visualization of outcomes, especially in the context of microarray cancer data analysis.

Once again, we extend our gratitude for your time and expertise in reviewing our work. Your input has been invaluable, and we will diligently address your suggestions to enhance the overall quality of the manuscript.

One critical aspect that still requires substantial improvement is the literature review. The current literature review appears limited in scope and depth. It is essential to expand this section to include a more extensive survey of related works in the field of high-dimensional optimization and microarray data analysis. A robust literature review will not only provide a broader context for the proposed algorithm but also help identify gaps and opportunities for future research.
- B, N., & V, I. (2022). Enhanced machine learning based feature subset through FFS enabled classification for cervical cancer diagnosis. International Journal of Knowledge-Based and Intelligent Engineering Systems, 26, 79–89. https://doi.org/10.3233/KES-220009
- Mohammed, M. S., Rachapudy, P. S., & Kasa, M. (2021). Big data classification with optimization driven MapReduce framework. International Journal of Knowledge-based and Intelligent Engineering Systems, 25(2), 173-183.

We appreciate your input and have taken your suggestion seriously. In response to your feedback, we have substantially expanded the literature review section in the manuscript, as you can see on lines 125 to 156. We have conducted a more comprehensive survey of related works in the fields of high-dimensional optimization and microarray data analysis. This expansion not only provides a broader context for our proposed algorithm but also helps identify gaps and opportunities for future research in a more robust manner.

As per your recommendation, we have expanded the literature review section and also included the two references you mentioned. These additional references provide valuable context to our work and contribute to a more comprehensive overview of the relevant literature.

We hope that the inclusion of these references aligns with your expectations and further strengthens the background and context for our research.

While the paper has improved in terms of clarity, some mathematical notations and equations can still be challenging to follow. It would be beneficial to simplify complex equations, provide clearer explanations, and possibly offer more intuitive examples to aid in comprehension.

We've expanded the explanations of mathematical notations and equations on pages 235 to 253, aiming to enhance clarity. Additionally, we've included more intuitive examples to aid comprehension. We appreciate your valuable input in improving our work.

To strengthen the paper's credibility, the authors should consider including a more extensive validation process, including comparisons with other state-of-the-art optimization algorithms. This would help demonstrate the advantages and limitations of the proposed Hybrid Whale Algorithm more effectively.

We fully understand the significance of a comprehensive validation process, including comparisons with other state-of-the-art optimization algorithms. While we acknowledge the value of such comparisons, we would like to highlight certain constraints we encountered during the preparation of this manuscript.

Firstly, the scope and length of our paper are already substantial, and we were conscious of providing a balanced level of detail and clarity. Given the space constraints in the journal, we opted to focus on providing a thorough presentation of our proposed Hybrid Whale Algorithm, its application to microarray cancer data, and detailed empirical results.

Secondly, conducting extensive comparisons with other optimization algorithms can be a resource-intensive endeavor, requiring a significant amount of computational resources and time. Unfortunately, due to limitations in both time and computational capacity, we were unable to perform a comprehensive benchmark against all relevant algorithms.

However, we are committed to addressing this aspect in future research endeavors and believe it would be a valuable extension of our work. In the current manuscript, we have placed significant emphasis on explaining the methodology, providing detailed results, and illustrating the algorithm's performance in the context of microarray cancer data.

We genuinely appreciate your insightful feedback, which will guide our future research directions, including more extensive validation and comparative analyses.

Once again, we thank you for your time and expertise in reviewing our manuscript.

The paper could benefit from improved visual presentation, such as the use of charts, graphs, and tables to illustrate key points and results. Visual aids can enhance the reader's understanding and engagement with the material.

We've already included charts, graphs, and tables in the paper to illustrate key points and results, enhancing the reader's understanding and engagement with the material. We appreciate your suggestion and are pleased to inform you that these visual aids are an integral part of our manuscript.

**Response to Reviewer #6:**

Please include the links to all data used in the study apart from carcinoma data, or perhaps their accession numbers for easy identification, the link szu.edu.cn is not enough to locate the data easily.

Thank you for your query regarding the availability of the data used in our study. We appreciate your thorough review and would like to address your concern.

In our study, we utilized data from various sources, including the carcinoma data, which we have previously provided. Additionally, for the remaining data used in our research, we sourced it from <https://csse.szu.edu.cn/staff/zhuzx/datasets.html>, as noted in our manuscript on page no. 581. To access the specific data sets used in our study, readers can follow the link provided, where they will find detailed information on each data set, including accession numbers, descriptions, and any relevant documentation. We believe that this link offers a comprehensive and user-friendly resource for accessing the data we utilized in our research.

It would be good if the authors added how the features used to train the SVM model were encoded for easy reproducibility.

Thank you for your suggestion regarding the encoding of features for our SVM model. We have provided a detailed explanation of this process in our manuscript, specifically in lines 495 to 517. This section offers a step-by-step guide for feature encoding, ensuring transparency and reproducibility.

**Response to Reviewer #7:**

Should Explain in detail About RESHWOA Algorithm and explain clearly how it works for the medical data.

For detailed insight into the RESHWOA algorithm, we have provided an extensive explanation spanning pages 217 to 253. In this section, we delve into the intricacies of the recombinative evolutionary strategy, outlining its core principles, mechanisms, and its specific role within the RESHWOA framework.

Furthermore, for a comprehensive understanding of RESHWOA as a whole, we have dedicated pages 307 to 333 to explain the algorithm's operation in detail. This section offers step-by-step insights into how RESHWOA functions, including its interactions with the data and optimization processes.

In our study, we address the crucial task of medical diagnosis, where the SVM (Support Vector Machine) classifier plays a pivotal role. The SVM classifier is highly valuable in diagnosing specific disorders because it can effectively distinguish between different classes or conditions. However, to harness the full potential of the SVM, we must first fine-tune its parameters to achieve the best possible performance, often measured by minimizing the Mean Squared Error (MSE).

One of the challenges we encounter in medical data analysis is the high dimensionality of the data. Medical datasets often comprise a large number of features or variables, which can introduce issues such as noise, redundancy, and the curse of dimensionality. To address these challenges and extract meaningful information from high-dimensional data, we employ data reduction techniques.

Our data reduction techniques are applied to both cancer data and normal data. By reducing the dimensionality of the dataset, we aim to make it more manageable while preserving the essential information necessary for accurate diagnosis.

Subsequently, we proceed with optimizing the SVM parameters based on the reduced data. This optimization step is critical to fine-tune the SVM model specifically for our dataset, ensuring optimal performance and minimal MSE. It allows us to tailor the SVM to the unique characteristics of the medical data under study.

Furthermore, we incorporate the recombinative evolutionary technique (RESHWOA) into our approach. This technique is noteworthy because it introduces diversity into the initial population. Diverse populations are advantageous in optimization problems as they explore a broader solution space, potentially leading to better solutions.

In summary, our approach combines data reduction techniques, SVM parameter optimization, and the inclusion of the recombinative evolutionary technique (RESHWOA) to address the challenges of medical data analysis. By optimizing the SVM model and introducing diversity in the initial population, we enhance the accuracy and robustness of our diagnosis process.

**Response to Reviewer #8:**

The authors have provided thoughtful and comprehensive responses to my comments, leaving me thoroughly satisfied.

We appreciate your kind words and are delighted to hear that you found our responses satisfactory. Your feedback and insights have been invaluable in improving the quality and clarity of our work.

**Response to Reviewer #9:**

The main strength of the RESHWOA algorithm is its ability to improve the diversity of the initial population of the WOA algorithm. This can help to prevent premature convergence and improve the performance of the algorithm. The algorithm is easy to implement and understand, which makes it a good choice for practitioners who are new to metaheuristic algorithms.

Thank you for highlighting the strengths of the RESHWOA algorithm in your feedback. We are pleased to hear that you recognize its ability to enhance the diversity of the initial population within the WOA algorithm, preventing premature convergence and improving overall performance. Additionally, we appreciate your observation that the algorithm's ease of implementation and understanding makes it a valuable choice, especially for practitioners new to metaheuristic algorithms.

Your positive assessment of these strengths aligns with our objectives in developing the RESHWOA algorithm, and we are encouraged by your feedback.

Consider adding a section comparing RESHWOA with other state-of-the-art algorithms(beside WOA) if possible.

We fully understand the importance of comprehensive validation, including comparisons with state-of-the-art optimization algorithms. However, due to constraints in the scope, length, computational resources, and time during the preparation of this manuscript, we focused on presenting our Hybrid Whale Algorithm, its application to microarray cancer data, and detailed empirical results. We acknowledge the limitations in not conducting extensive comparisons with other algorithms but express our commitment to addressing this in future research. We value the reviewer's feedback and appreciate their guidance for our future endeavors.

While passive voice is common in scientific writing, overuse can make the text harder to read. Consider using active voice where it improves clarity.

Thank you for your valuable feedback regarding the use of passive voice in our manuscript. We appreciate your input, and we have taken your suggestion to heart.

Upon careful review, we have made concerted efforts to address this issue and have revised the manuscript to incorporate active voice where it enhances clarity without compromising scientific rigor. We believe that these changes have significantly improved the readability and overall quality of the text.

We genuinely appreciate your constructive feedback, which has played a crucial role in enhancing the clarity and coherence of our work.
